# Supplementary material for: The effect of antenatal care on perinatal outcomes in Ethiopia: A systematic review and meta-analysis
Source: PLoS One. 2021 Jan 14;16(1):e0245003. doi: 10.1371/journal.pone.0245003 (PMC7808692; doi:10.1371/journal.pone.0245003)
Supplement: S1 Table — (DOCX) [file pone.0245003.s001.docx]

**S1 Table. Searching using MEDLINE via PubMed**

| S.no | Searching | Results |
| --- | --- | --- |
| 1 | ((((("antenatal care"[All Fields] OR ("prenatal care"[MeSH Terms] OR ("prenatal"[All Fields] AND "care"[All Fields]) OR "prenatal care"[All Fields])) OR "maternity care"[All Fields]) OR ("obstetrics"[MeSH Terms] OR "obstetrics"[All Fields])) OR "maternal health care"[All Fields]) OR ("maternal health services"[MeSH Terms] OR ("maternal"[All Fields] AND "health"[All Fields] AND "services"[All Fields]) OR "maternal health services"[All Fields])) OR "pregnancy care"[All Fields] | 284394 |
| 2 | ((((((((("perinatal outcomes"[All Fields] OR ("perinatal mortality"[MeSH Terms] OR ("perinatal"[All Fields] AND "mortality"[All Fields]) OR "perinatal mortality"[All Fields] OR "perinatal death"[MeSH Terms] OR ("perinatal"[All Fields] AND "death"[All Fields]) OR "perinatal death"[All Fields] OR ("perinatal"[All Fields] AND "mortality"[All Fields]))) OR ("perinatal death"[MeSH Terms] OR ("perinatal"[All Fields] AND "death"[All Fields]) OR "perinatal death"[All Fields])) OR ("stillbirth"[MeSH Terms] OR "stillbirth"[All Fields])) OR "early neonatal death"[All Fields]) OR "early neonatal mortality"[All Fields]) OR ("foetal death"[All Fields] OR "fetal death"[MeSH Terms] OR ("fetal"[All Fields] AND "death"[All Fields]) OR "fetal death"[All Fields])) OR "newborn death"[All Fields]) OR "newborn mortality"[All Fields]) OR ("infant mortality"[MeSH Terms] OR ("infant"[All Fields] AND "mortality"[All Fields]) OR "infant mortality"[All Fields])) OR ("infant death"[MeSH Terms] OR ("infant"[All Fields] AND "death"[All Fields]) OR "infant death"[All Fields]) | 454504 |
| 3 | Ethiopia | 46429 |
|  | 1 AND 2 AND 3 | 6341 |
|  | Publication date from 1990/01/01 to 2020/06/30 | 6238 |

**S2 Table. Searching using EMBASE (via Ovid)**

| s.no | Searching | Result |
| --- | --- | --- |
| 1 | antenatal care.mp. or prenatal care/ or prenatal care.mp. or prenatal care/ or maternal health care.mp. or maternal care/ or maternity care.mp. or maternal care/ or maternal care/ or pregnancy/ or prenatal care/ or pregnancy care.mp. | 762927 |
| 2 | perinatal mortality.mp. or perinatal mortality/ or perinatal death.mp. or perinatal mortality/ or perinatal death/ or fetus death/ or fetal death.mp. or fetus death/ or newborn mortality/ or perinatal mortality/ or early neonatal mortality.mp. or newborn death/ or stillbirth.mp. or stillbirth/ or newborn mortality.mp. or newborn mortality/ or newborn death.mp. or newborn death/ or perinatal outcomes.mp. or pregnancy outcome/ | 138373 |
| 3 | Ethiopia.mp. or Ethiopia/ | 19276 |
| 4 | 1 and 2 and 3 | 241 |
|  | limit 4 to (human and english language and yr="1990 -Current") | 215 |

**S3 Table. Searching using CINAHL.**

| Search ID | Search Terms | Result |
| --- | --- | --- |
| 1 | antenatal care OR prenatal care OR maternal health care OR maternity care OR pregnancy care | 36,542 |
| 2 | perinatal mortality OR perinatal death OR fetal death OR early neonatal mortality OR stillbirth OR newborn mortality OR newborn death OR perinatal outcomes | 25,264 |
| 3 | Ethiopia | 5,570 |
| 4 | 1 AND 2 AND 3 | 82 |
| 5 | Narrow by Language: - english | 82 |
| 6 | Limiters - Published Date: 19901201-20200531; Human | 47 |
