# Supplementary material for: The effect of antenatal care on perinatal outcomes in Ethiopia: A systematic review and meta-analysis
Source: PLoS One. 2021 Jan 14;16(1):e0245003. doi: 10.1371/journal.pone.0245003 (PMC7808692; doi:10.1371/journal.pone.0245003)
Supplement: S2 Table — (DOCX) [file pone.0245003.s002.docx]

**S4 Table. Assessment of risk of bias for individual study (RoBANS).**

| **Study ID** | **Bias in selection of participants into the study** | **Bias due to missing/ incomplete data** | **Performance bias occurs during measurement of exposure** | **Detection bias due to not blinding outcomes assessment** | **Bias due to confounding variables** | **Bias in selection of the reported results** |
| --- | --- | --- | --- | --- | --- | --- |
| Adane et al. 2014 | Low  This study included all women who gave birth throughout the day and night during the one month study period. | Low  There is less non-response rate. No information is reported about missing data. | Low  Data were collected using a structured questionnaire. The outcome measure was unlikely to be influenced. | High  No information | Low  Potential confounders were considered in relation to antenatal care e.g. socio-economic factors and obstetric characteristics. | Low  The expected outcome (stillbirth) was reported. Unlikely to be manipulated. |
| Ballard et al. 2016 | Low  A randomly selected sample of women in northern and eastern Ethiopia who had delivered a neonate in the preceding 12 months completed. Data were obtained between May and December 2014. | Low  No effect on the outcome. No missing data. | Low  The adapted Haddad 20-item scale, which has been validated in countries with similar health systems to Ethiopia, was used | High  Not mentioned | High  Potential confounders were not controlled for prenatal care. Reliability and validity of measurement of an important domain was low and residual confounding are expected. | Low  The stillbirth was reported as expected outcome and unlikely to be manipulated. |
| Berhan 2014 | Low  All women who were admitted and managed in Hawassa University Hospital between January 2006 and December 2012 were selected retrospectively. | Low  Cases were excluded because of incomplete documentation. No missing data reported. | Unclear  The author didn’t mention how measurement of exposure were done. | High  No information | High  Confounding like mothers’ age, mothers’ residence, distance traveled in km, educational status were not controlled. | Low  The survival of babies in the perinatal period were taken as expected primary outcome indicators and unlikely to be manipulated. |
| Berhie and Gebresilassie 2016 | Low  All women age 15–49 and all men age 15–59 in these households were eligible for individual interview from September 2010 through June 2011 with a nationally representative sample of nearly 18,500 households were selected. | Low  Adjusting for the missing data have been done before the analysis. | Low  The nationally validated tool of Ethiopian Demographic and Health Survey was used. The outcome measure was unlikely to be influenced. | High  No information | Low  Among the factors, region of residence, maternal age, place of residence, education level, body mass index (BMI) etc. were seen for confounding effects. | Low  Stillbirth was the expected outcome of the study. Unlikely to be manipulated during reporting. |
| Eyob and Worku 2003 | Low  All deliveries included were between 28 weeks and 42 weeks of gestational age. The study was carried out from l^st^ January 1995 to December 31, L996 | Low  No missing data. | Unclear  Measurement was not clearly mentioned in the study. | High  No information | High  The potential confounding variable of prenatal care were not controlled. | Low  Stillbirth was expected outcome and unlikely to be manipulated. |
| Getiye and Fantahun 2017 | Low  All deliveries conducted from January 1/ 2014-Dec 31/ 2014 were used as a source population. These hospitals were selected based on availability of both delivery and neonatal intensive care unit service during the time of data collection. | Low  The response rate was high and no missing data. | Low  Data was collected using structured questionnaire from medical records of mothers and neonates. The outcome measure was unlikely to be influenced. | High  No information | Low  Potential confounders i.e. education, obstetric complications, gestational age, mode of delivery etc. were controlled. | Low  Perinatal mortality was clearly explained as expected outcome and unlikely to be manipulated. |
| Goba et al. 2017 | Low  The case group was recruited consecutively until the sample size was reached and the control group was selected from all potentially eligible patients using systematic sampling from women who delivered at 20 health centers and hospitals in the Southern Zone of Tigray, Ethiopia, from February 1 to September 30, 2016. | Low  No missing data. | Unclear  The authors didn’t describe the measurements. | High  There was no information whether the authors controlled detection bias. | Low  Confounders like maternal age, distances from health facility and education were controlled. | Low  Perinatal mortality was expected outcome and unlikely to be manipulated. |
| Lakew et al. 2017 | Low  The survey was designed to produce representative estimates for the country as a whole, for the urban and the rural areas one at a time, and for each of the 11 regions. | Low  No missing data. | Low  These instruments adopted from the 2011 Ethiopia DHS based on the requirements of clients of the Ethiopian mini demographic health survey. The outcome measure was unlikely to be influenced. | High  There was no information given. | Low  Confounders like socio-demographic and economic characteristics and obstetrics related characteristics were controlled. | Low  The response variable for this study was stillbirth outcomes. |
| Roro et al. 2018 | Low  All perinatal deaths occurring between March 2011 and December 2012 were taken as cases. For every perinatal death, two controls (newborns from mothers included in the cohort who survived the perinatal period) were randomly selected by using a lottery method from the frame of the cohort. | Unclear  Since 55 (1.2%) of the mothers were lost to follow-up and their pregnancy outcome are unknown. | Low  Data was collected using the World Health Organization verbal autopsy questionnaire for neonatal death after adapting it to the local context. The outcome measure was unlikely to be influenced. | High  No information | Low  Potential confounders like maternal age, marital status, education status and occupation were controlled. | Low  Perinatal death was the outcome variable. |
| Tilahun & Assefa 2017 | Low  Participant mothers were identified using systematic random sampling until required numbers of sample obtained. | Low  The response rate was high and there was no missing data. | Low  The data were collected using structured questionnaires and check lists which were adapted from similar survey. The outcome measure was unlikely to be influenced. | High  No information. | Low  Confounders were controlled. | Low  The still birth was expected outcome variable. |
| Tilahun and Gaym 2008 | Low  All parous women, with singleton pregnancy, and who fulfilled the selection criteria were included in the study. | Unclear  Missing data and non-response were not mentioned clearly. | Low  Used a structured questionnaire. The outcome measure was unlikely to be influenced by knowledge of the intervention (ANC follow up) received by study participants. | High  No information. | Low  Maternal age, residence, education were controlled as confounding variables. | Low  The expected outcome was perinatal mortality. |
| Welegebriel et al. 2017 | Low  For each case 3 controls who were survived births were selected, which were born immediately preceding or following a case. | Unclear  No missing data explained and there was few non-response. | Low  Data extraction checklist was adapted from related surveillance hence, the outcome measure was unlikely to be influenced. | High  No information | Low  The confounding effect of age of the women, ANC visit, gravidity, obstructed labour, antenatal risks, labour abnormality, length of labor, and the weight of child were seen. | Low  Still birth was expected outcome. |
| Worede and Dagnew 2019 | Low  All mothers who had encountered stillbirth were recruited as cases consecutively until the required sample size was reached and four controls per case were selected using a systematic sampling technique. | Low  Ono-response was considered and no missing data. | Low  A pretested, structured questionnaire and checklist was used. The outcome measure was unlikely to be influenced | High  Information was not given. | Low  Confounder like education was controlled. | Low  The dependent variable was stillbirth. |
| Worku et al. 2013 | Low  All pregnant women in the selected kebeles were identified by a baseline survey and joined the follow up scheme. Then new pregnant women were added through a monthly detection until the sample size was adequate for the analysis. | Low  Only 4.7% attrition. | Low  Data was collected using a follow up questionnaire, which was prepared for a progressive evaluation. The outcome measure was unlikely to be influenced | High  No information | Unclear  There is no information whether the authors measured/controlled confounding variables of exposure variable. | Low  Perinatal deaths was expected as outcome variable. |
| Chekol A., 2011 | Low  Study participants were admitted to the hospital for delivery within the study period and mothers, who were willing to participate voluntarily and able to communicate, were included in the study. | Low  No attrition and the study have high response rate. | Low  The data collection tool used was a structured pretested questionnaire prepared in local language and bias is unlikely. | High  Not mentioned in the study. | Low  The authors have performed multivariable analysis to control confounding. | Low  The outcome is defined in the study clearly. |
| Aragaw Y., 2016 | Low  The survey was conducted among all women who gave birth in labor and maternity ward and bias is unlikely. | Low  There is high response rate and no attrition. | Low  A structured checklist was used to collect data and bias is unlikely. | High  Not mentioned in the study. | Low  The authors controlled the confounding variables in the study. | Unclear  The authors not defined the outcome. |
| Mihiretu A. et al, 2017 | Low  The study included all women who gave birth study setting and bias is unlikely. | Low  The study has hundred response rate and less likely to have attrition bias. | Low  A pretested structured checklist was used to collect data and measurement bias is unlikely. | High  Not mentioned in the study. | Low  There were controlled confounding variable in the study and bias is unlikely. | Unclear  Outcome variable was not defined. |
