# Supplementary material for: The effect of antenatal care on perinatal outcomes in Ethiopia: A systematic review and meta-analysis
Source: PLoS One. 2021 Jan 14;16(1):e0245003. doi: 10.1371/journal.pone.0245003 (PMC7808692; doi:10.1371/journal.pone.0245003)
Supplement: S3 Table — (DOCX) [file pone.0245003.s003.docx]

**S3 Table. Searching using CINAHL.**

| Search ID | Search Terms | Result |
| --- | --- | --- |
| 1 | antenatal care OR prenatal care OR maternal health care OR maternity care OR pregnancy care | 36,542 |
| 2 | perinatal mortality OR perinatal death OR fetal death OR early neonatal mortality OR stillbirth OR newborn mortality OR newborn death OR perinatal outcomes | 25,264 |
| 3 | Ethiopia | 5,570 |
| 4 | 1 AND 2 AND 3 | 82 |
| 5 | Narrow by Language: - english | 82 |
| 6 | Limiters - Published Date: 19901201-20200531; Human | 47 |
