# Supplementary material for: The effect of antenatal care on perinatal outcomes in Ethiopia: A systematic review and meta-analysis
Source: PLoS One. 2021 Jan 14;16(1):e0245003. doi: 10.1371/journal.pone.0245003 (PMC7808692; doi:10.1371/journal.pone.0245003)
Supplement: Attachment — Submitted filename: Response to Reviewers.docx [file pone.0245003.s007.docx]

**To: PLOS ONE Journal**

**Subject: Revision and resubmission of manuscript (manuscript Number:** PONE-D-20-22748R1**)**

**Dear Editors and reviewers**

We thank you very much for valuable comments on our manuscript entitled “**The effect of antenatal care on perinatal outcomes in Ethiopia: A systematic review and meta-analysis**”. The comments and suggestions offered by the reviewer have enormously helped us to improve our manuscript. We have considered each and every comment and responded point by point, indicating how we addressed them and showing the changes we made. The changes are presented in the revised manuscript. Our responses to the reviewer’s comments are provided below. **Furthermore, we would like to inform you that our article will be published for free if accepted by your journal.**

We hope the revised manuscript is better suited to your esteemed journal and we are happy to consider any further revision as deemed necessary.

Sincerely

Kasiye Shiferaw

Response to reviewers

| Comments | Response |
| --- | --- |
| Reviewer #1 |  |
| The authors of this manuscript have addressed many of the comments satisfactorily and overall the manuscript has a nice flow with improved clarity of writing. However, there are still syntax, punctuation and spelling errors (minor concern that is easily corrected), | The comments were corrected in the main documents. Thank you. |
| but more importantly, the authors continue to conflate perinatal mortality, neonatal mortality and stillbirths. | We found your comment astonishing. We learnt a lot from you frankly. We defined these terms in the operational definition part. As you said these terms are not the same. We cautiously analyzed these findings in current version of the manuscript. We have explained this concern in detail below on response we provided to specific comment #4. |
| Of note - WHO and UNICEF The Every Newborn Action Plan: Goals for reducing newborn mortality and preventing stillbirths: Goal 1. End preventable newborn deaths. By 2030, all countries will have reached the target of 12 or less newborn deaths per 1000 live births and will continue to reduce death and disability, ensuring that no newborn is left behind. Goal 2. Ending preventable stillbirths By 2030, all countries will have reached the target of 12 or fewer stillbirths per 1000 total births and will continue to improve equity. OR (updated), Goal 1. End preventable newborn deaths. By 2035, all countries will reach the target of 10 or less newborn deaths per 1000 live births and continue to reduce death and disability, ensuring that no newborn is left behind. Goal 2. Ending preventable stillbirths By 2035, all countries will reach the target of 10 or less stillbirths per 1000 total births and continue to close equity gaps. | Thank you, it is nice recommendation. We took goal 1 End preventable newborn deaths. By 2030, all countries will have reached the target of 12 or less newborn deaths per 1000 live births and will continue to reduce death and disability, ensuring that no newborn is left behind in our manuscript as we explained it in the current version. We agreed all goals are reducing the newborn death and making sure all countries are achieving the goal. |
| Specific comments: |  |
| 1. In the Introduction section: Lines 68-69 and line 228. It would be helpful for international readers if the “focused ANC model” referenced a few times in the manuscript is explained. | Thank you for the comment. We defined it as ‘**Focused ANC model** is four visits providing essential evidence based interventions – a package to achieve the full life-saving potential that ANC promises for women and babies.’ |
| 2. In the Introduction section: Line 70. “Reduction in the gaps reductions” – needs to be clarified. | The comment was accepted. Thank you. The repeated phrase was removed in the new version of the manuscript. We rewrote the sentence. |
| 3. In the Methods section: Line 129. Authors appear to suggest that an attempt was made to obtain missing data from its source, but not always successful – authors should add a statement as to how missing data was handled in the analysis. | As you know missing data is challenging most of the time. We tried to request for further information when we thought the data lack clarity and/or missing and unfortunately there was no study which was excluded due to clarity and/or missing data. |
| 4. In the Methods section: Lines 174-178. Authors should separate newborn deaths and stillbirths. Hence, clarify and probably show numbers used as numerator and denominator for each of the following: Stillbirth rate per 1000 total births = number of stillbirths/total number of births, whereas neonatal mortality rate per 1000 live births = number of neonatal deaths/total number of live births. As currently stated in the manuscript, there is an unresolved discrepancy in reporting. See also Line 231. | Thank you for the comments. Stillbirth rate is calculated using numerator of number of stillbirths and denominators of number of births (dead or alive) whereas early neonatal mortality is calculated using numerator of neonatal death and denominator of live birth during 7 days of life.  Number of stillbirths per 1000 births (live and stillbirths). It was 38 per 1000 total births (stillbirths and live births) in our review.  Early neonatal mortality rate is the death of neonate per 1000 live births within 7 days of life. The rate was 19 per 1000 live birth after correction was made based on your recommendation.  Similarly, the denominator used for perinatal mortality rate (PMR) determination was total births after 28 weeks of gestation. Since the majority of the studies included reported PMR as per 1000 total births, data presented in some primary studies with a denominator of total live births were changed into total births by determining new PMR taking into account the reported total deliveries, total still births and total early neonatal deaths. Therefore, the perinatal mortality rate in this finding was 41 per 1000 total births (total deliveries, total still births and total early neonatal deaths) as we mentioned in the previous version. |
| 5. In the Results section: Line 248-249. “…58% and 66% lower risk of perinatal mortality and stillbirth among women who attended at least one ANC..” – these percentages may need to be modified after recommendations in Comment #4. | Thank you for the comments. There is no any relationship between these percentages and definitions of these terms (comments #4). These percentages were taken from risk ratio which were found from random effect model calculation. Hence the risk ratio for perinatal mortality and stillbirths were 0.42 and 0.34 making 58% (1-0.42) and 66% (1-0.34) percentages. We did this because experts recommend explaining finding in percentage, rather putting as obsolete number. |
